# *Ex vivo* and *in vivo* HIV-1 latency reversal by “Mukungulu,” a protein kinase C-activating African medicinal plant extract

**DOI:** 10.1128/mbio.03816-24

**Published:** 2025-04-23

**Authors:** Khumoekae Richard, Zhe Yuan, Hsin-Yao Tang, Aaron R. Goldman, Riza Kuthu, Boingotlo Raphane, Emery T. Register, Paridhima Sharma, Brian N. Ross, Jessicamarie Morris, David E. Williams, Carol Cheney, Guoxin Wu, Karam Mounzer, Gregory M. Laird, Paul Zuck, Raymond J. Andersen, Sundana Simonambango, Kerstin Andrae-Marobela, Ian Tietjen, Luis J. Montaner

**Affiliations:** 1 The Wistar Institute 36586, Philadelphia, Pennsylvania, USA; 2 University of Botswana 54547, Gaborone, Botswana; 3 Departments of Chemistry and Earth, Ocean and Atmospheric Sciences, University of British Columbia 8166, Vancouver, British Columbia, Canada; 4 Merck and Co Inc 2793 , Rahway, New Jersey, USA; 5 Jonathan Lax Immune Disorders Treatment Center, Philadelphia Field Initiating Group for HIV-1 Trials, Philadelphia, Pennsylvania, USA; 6 AccelevirDx, Baltimore, Maryland, USA; 7 Kwame (Legwame) Traditional Association, Mmadinare, Botswana; University of California, Davis, California, USA

**Keywords:** HIV, latency reversal, natural products, traditional medicines, *ex vivo*, humanized mice

## Abstract

New HIV latency-reversing agents (LRAs) are needed that can reactivate and/or eliminate HIV reservoirs. “Mukungulu,” prepared from the plant *Croton megalobotrys* Müll Arg., is traditionally used for HIV/AIDS management in northern Botswana despite an abundance of protein kinase C-activating phorbol esters (“namushens”). Here, we show that Mukungulu is tolerated in mice at up to 12.5 mg/kg while robustly reversing latency in antiretroviral therapy (ART)-suppressed HIV-infected humanized mice at 5 mg/kg. In primary cells from ART-suppressed people living with HIV-1, 1 µg/mL Mukungulu reverses latency at levels similar to or superior to anti-CD3/CD28 positive control, based on HIV gag-p24 protein expression, while the magnitude of HIV reactivation in peripheral blood mononuclear cells corresponds to intact proviral burden in CD4+ T-cells. Bioassay-guided fractionation identifies five namushen phorbol esters that can reactivate HIV, but when combined, they do not match Mukungulu’s activity, suggesting the presence of additional enhancing factors. Together, these results identify Mukungulu as a robust natural LRA that is already in use by humans and which may warrant inclusion in future HIV cure and ART-free remission efforts.

**IMPORTANCE:**

Current HIV therapies do not act on the latent viral reservoir, which is the major obstacle toward achieving a drug-free HIV remission and/or an HIV cure. “Mukungulu,” a bark preparation from *Croton megalobotrys* Müll Arg., has been documented for its traditional use for HIV/AIDS management in northern Botswana. Here, we show that Mukungulu activates viral reservoirs, a key step toward identifying and potentially eliminating these reservoirs, in both cells from people living with HIV as well as in HIV-infected humanized mice. The majority of this activity is due to the abundance of five phorbol esters (“namushens”). This reverse pharmacology-based approach has therefore identified a potent activator of viral reservoirs that is already traditionally used by humans, which in turn can inform and advance western HIV cure and drug-free remission efforts.

## INTRODUCTION

While antiretroviral therapy (ART) is a remarkable milestone that has reduced HIV/AIDS-related morbidities and mortalities globally, ART is not curative. A major obstacle to HIV eradication is the presence of inducible, replication-competent proviral DNA that persists in cellular reservoirs, particularly in CD4+ T-cells, which can reactivate at any time to produce infectious virus (1, 2). Because of this, people living with HIV (PLWH) must remain on ART for life. Long-term use of ART is also increasingly linked to non-AIDS co-morbidities including renal disorders, cancer, and cardiovascular diseases, potentially due to residual viral antigen production, ongoing immune activation, and/or chronic inflammation (3–5). As a result, new therapies that can support lowering the viral burden on ART by targeting viral reservoirs continue to be needed.

**Fig 1:**
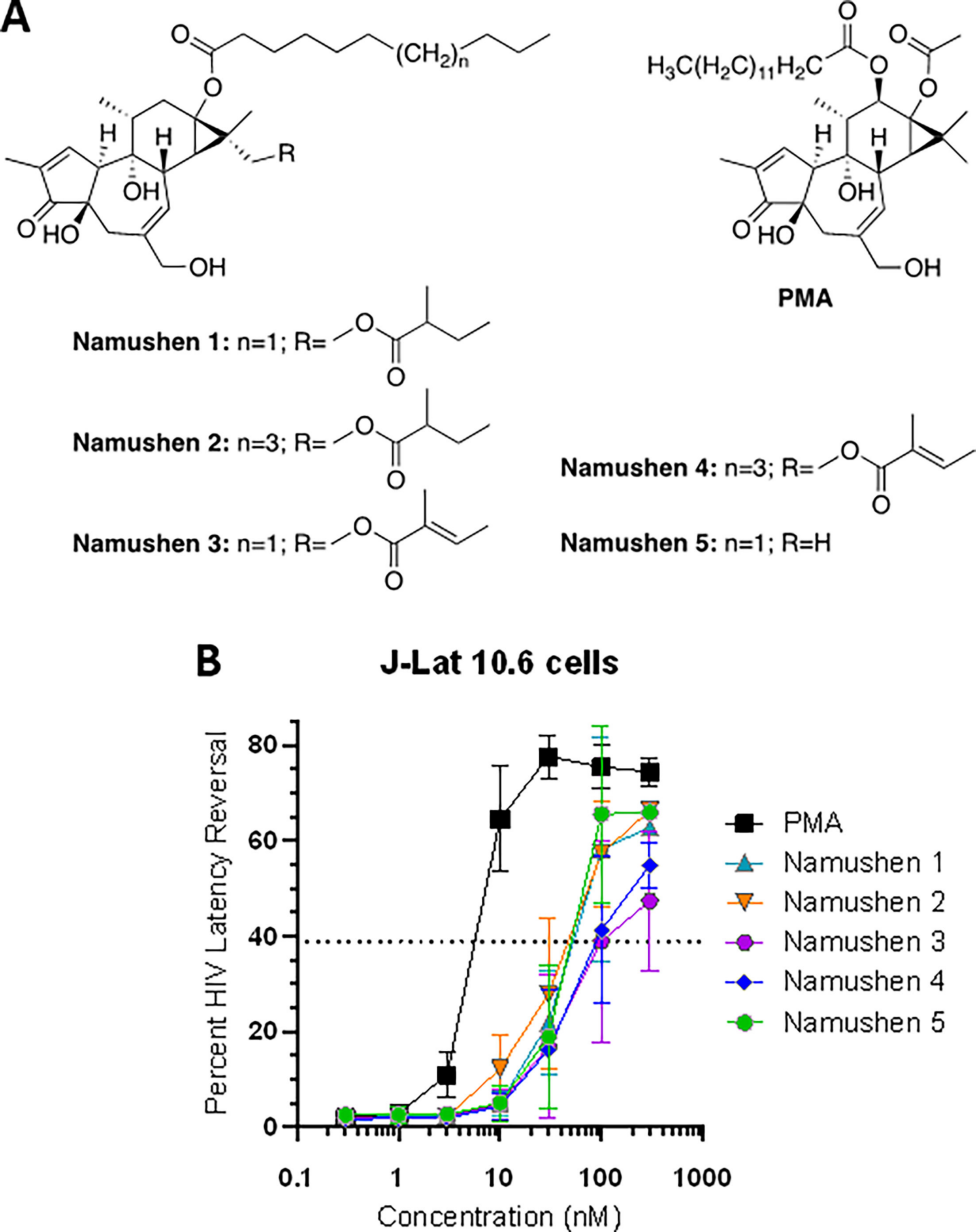
Isolation of namushen phorbol esters as major LRA components of crude Mukungulu extract. (**A**) Structures of five isolated namushen phorbol esters and PMA. (**B**) Effects of namushens and control phorbol ester PMA on HIV latency reversal in J-Lat 10.6 cells.

One important strategy to identify and eliminate HIV reservoirs involves the use of latency-reversing agents (LRAs) that activate HIV-1 expression (in the co-presence of ART to eliminate reservoir re-seeding). This viral expression, in combination with immunotherapy support, could then expose these latently infected cells to immune-mediated clearance, an approach colloquially known as “shock-and-kill” or “kick-and-kill” (6–8). Numerous LRAs have been identified encompassing different molecular mechanisms of action such as histone deacetylase inhibitors (HDACis), activators of protein kinase C (PKC) signaling, bromodomain and extra-terminal bromodomain inhibitors, DNA methyltransferase inhibitors, histone methyltransferase inhibitors, programmed cell death protein-1 inhibitors, toll-like receptor agonists, and noncanonical NF-κB agonists, among others (8). However, most of these LRAs remain poorly characterized in both *ex vivo* models using primary blood cells from PLWH and/or *in vivo* animal models (9–11). Furthermore, LRAs that have been tested to date in humans such as HDACis largely represent repurposed anti-cancer drugs with limited to no impact on viral reservoir size in PLWH (9–11). By contrast, in spite of greater activity *in vitro*, the large majority of PKC activators have not advanced beyond *ex vivo* models due to the risk that single PKC activator compounds, for example, phorbol esters like phorbol 12-myristate 13-acetate (PMA; Fig. 1) or prostratin, may cause widespread T-cell activation and *in vivo* toxicity (12, 13).

Using a reverse pharmacology approach, medicinal plants have been documented that are traditionally used for HIV/AIDS management in Sub-Saharan Africa and elsewhere, including some with latency-reversing properties (14, 15). For example, we previously described the traditional use of *Croton megalobotrys* Müll Arg. bark preparations, locally called “Mukungulu,” to manage HIV/AIDS in northern Botswana (16, 17). Though Botswana introduced free and universal ART access in 2002, many PLWH initially hesitated to enroll in these programs due to perceived stigma and discrimination and instead chose to rely on local primary healthcare providers including traditional healers. We documented that Mukungulu is part of a three-step traditional treatment regimen that was offered to patients in northern Botswana during that time and is now also used as a supplement to standard ART (17). According to the healers, patients are treated if presenting with substantial weight loss, chronic diarrhea, fevers, skin infections or wounds, and/or lethargy. The first two steps of the treatment regimen consisted of separate plant preparations (from *Cassia siberiana* D.C. roots and *Vitex doniana* [Sweet] bark) taken over several weeks which also inhibit HIV replication *in vitro* (17). Mukungulu is then administered as a single dose (as the third step in the regimen), where the patient remains under close observation by the healer for 48–72 hours. After recovery, patients are instructed to continue the use of ART, obtain plasma viral load (pVL) results from a local clinic after 3 weeks, and not repeat Mukungulu treatment for at least 6–12 months (16, 17). Following treatment, patients report improved weight gain, recovery from chronic diarrhea, fewer fevers, wound healing, and increased energy for at least 3 months (16, 17). Notably, we found that crude Mukungulu extract reversed HIV latency in cell lines and contained phorbol esters (namushens 1 and 2; Fig. 1) that structurally resemble PMA and prostratin and were sensitive to PKC inhibition *in vitro*, supporting a mode of action involving PKC activation (16). These properties, together with Mukungulu’s traditional single dose following anticipated viral suppression, suggested it may function as a novel LRA.

To further investigate the potential of Mukungulu as an LRA acting through PKC activation, we describe here the latency-reversing properties of Mukungulu and its active components in total peripheral blood mononuclear cells (PBMCs) and isolated CD4+ T-cells from ART-suppressed PLWH. We also document Mukungulu’s activity when used in ART-suppressed HIV-infected humanized mice, where we observe robust latency reversal as well as *in vivo* tolerability.

## RESULTS

### Isolation of phorbol ester-class LRAs (“namushens”) from crude Mukungulu extract

We previously isolated two phorbol esters from Mukungulu (namushen 1 and 2, named for the healer who first communicated the traditional use of Mukungulu) that could reverse HIV latency *in vitro* (16, 17). Therefore, we began by expanding the characterization of Mukungulu extract to identify if any other active components apart from namushens 1 and 2 were present. To do this, we obtained 81.3 g of an oily dark brown CH_2_Cl_2_/methanol crude extract from a collection of *Croton megalobotrys* bark powder and subjected it to bioassay-guided fractionation using J-Lat 9.2 cells and previously described *in vitro* approaches (16, 18) (see Supplemental Material). Briefly, cells were treated with extracts, fractions, or compounds for 24 hours, and green fluorescent protein (GFP) reporter expression, a marker of provirus expression (19), was assessed by flow cytometry. Mukungulu crude extract was fractionated using a combination of silica gel columns and high-performance liquid chromatography (HPLC; see Supplemental Material). This approach led to re-isolation of namushens 1 and 2, as expected, but also three new phorbol ester species which we named namushens 3, 4, and 5 (Fig. 1A Table S1 and Fig. S1 to S6). Notably, no bioactive fractions were identified that lacked namushens, establishing that phorbol esters are the primary driver of *in vitro* HIV latency reversal in Mukungulu.

To reconfirm HIV latency reversal by purified namushens, we assessed their activity as compared to PMA in J-Lat 10.6 cells, which resemble J-Lat 9.2 cells except for a different genomic location of provirus integration. With this approach, we determined a half-maximal effective concentration (EC_50_) of 4.1 nM for PMA compared to EC_50_s of 48.0, 41.2, 135.2, 99.5, and 40.9 nM for namushens 1–5, respectively (Fig. 1B), indicating that namushens have roughly 10- to 30-fold lower activity than PMA, and the distal double bond of the hydrophobic tail may be a determinant of activity for namushens 3 and 4. These results confirm that namushen phorbol esters can mediate latency reversal when used in isolation from the parental crude extract.

### Namushen levels are broadly similar across separate Mukungulu crude extracts but do not recapitulate all Mukungulu latency reversal activity *in vitro*

**Fig 2:**
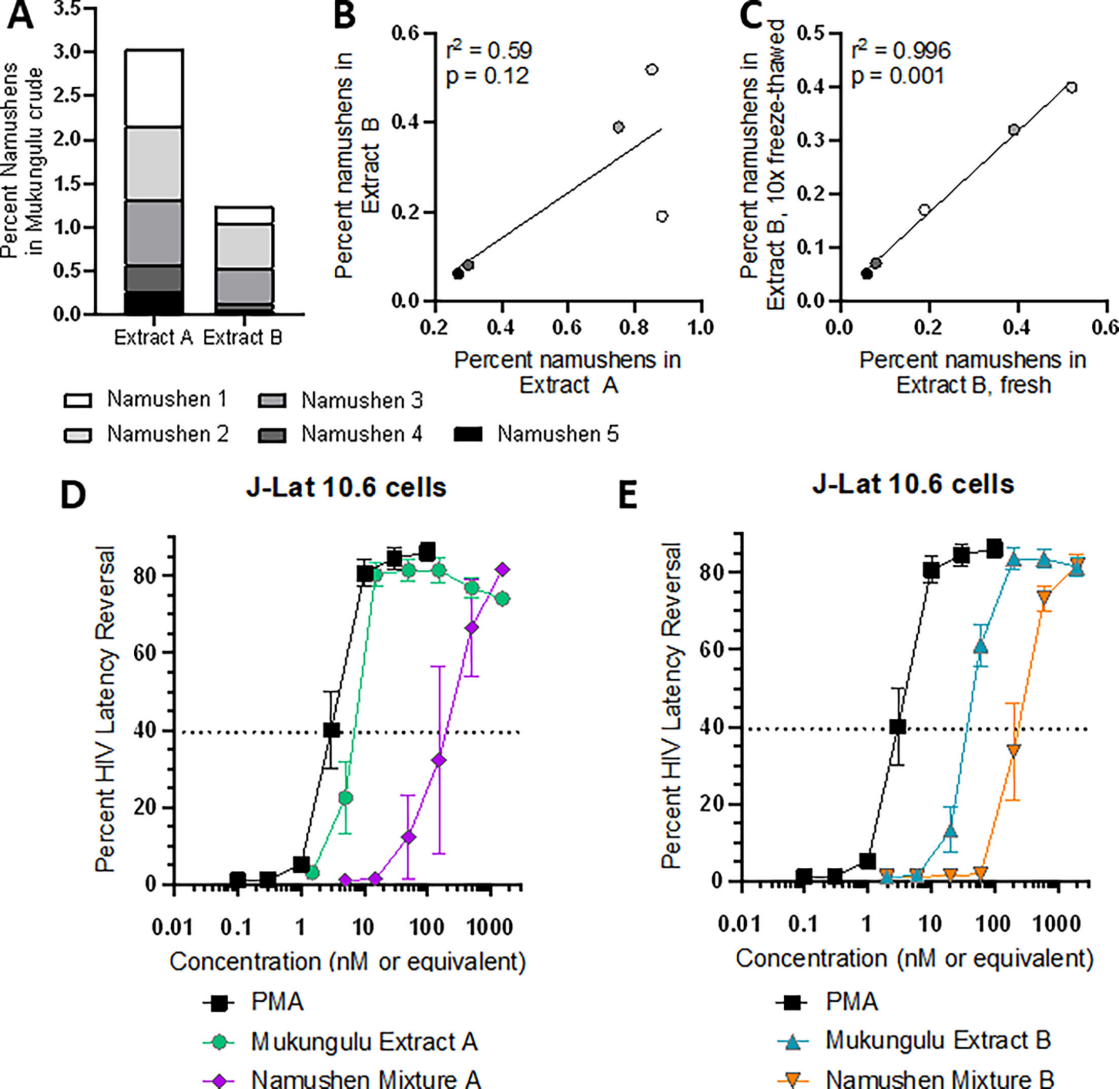
Namushen levels are broadly similar across different collections of Mukungulu medicinal plants but do not recapitulate total Mukungulu activity. (**A**) Steady-state polar metabolite analysis for namushens phytoconstituents in different Mukungulu crude extracts. (**B**) Correlation of namushen levels in Mukungulu extracts A and B. (**C**) Correlation of namushen levels of Mukungulu extract B either prepared fresh in DMSO or frozen and thawed in DMSO 10 times. (**D and E**) Effects of Mukungulu extract A (**D**) and extract B (**E**) on HIV latency reversal in J-Lat 10.6 cells when compared to namushens 1–5 recapitulated in equivalent molar concentrations to parental extracts.

To determine the relative abundance of namushens in crude Mukungulu, we next assessed namushen content level using liquid chromatography-mass spectrometry (LC-MS) analysis (Fig. 2). For this analysis, we investigated both the crude Mukungulu extract described above (extract A) as well as an independently prepared methanolic extract from Mukungulu collected from a separate location in a different year (extract B) (17). This analysis determined that namushens made up 3.1% of the total components in extract A, comprising approximately equal proportions of namushens 1, 2, and 3 and approximately 70% less of namushens 4 and 5 (Fig. 2A; Table S2). In extract B, namushens comprised 1.2% of total components, which consisted predominantly of namushen 2 followed by 3 and 1 (Fig. 2A). Simple linear regression analysis indicated a good correlation of respective namushen levels between extracts A and B (*r*^2^ = 0.59), although this did not reach statistical significance as measured by analysis of variance (ANOVA; *P* = 0.12; Fig. 2B). However, significant correlation of namushens was observed from a sample of extract B freshly resuspended in dimethyl sulfoxide (DMSO) compared to a sample of extract B in DMSO which was frozen and thawed 10 times (*r*^2^ = 0.996, *P* = 0.001; Fig. 2C), indicating good stability of namushens *in vitro*. These results indicate that namushens make up ~1%–3% of total soluble components of Mukungulu with proportions and amounts that are, to a first approximation, broadly consistent across independently sourced samples.

We next asked whether namushens 1–5 were sufficient to recapitulate the full LRA magnitudes observed in crude extracts. Therefore, we reconstituted the five namushens in the same proportions and concentrations to match their percentage of namushens to the total volume present in the parental extracts (i.e., identical to levels seen in Fig. 2A; Table S2). Namushen mixtures were then tested side-by-side with batch A or B for the ability to reverse latency in J-Lat 10.6 cells. As shown in Fig. 2D, treatment of J-Lat 10.6 cells with positive control PMA reversed latency with an EC_50_ of 1.0 nM, while Mukungulu extract A exhibited a relative EC_50_ of 2.7 nM (where 1 µg/mL of Mukungulu was calculated to contain 20.6 nM of namushens; Table S2). By contrast, namushen mixture A had an EC_50_ of only 130 nM, requiring a 48-fold higher concentration to achieve the same activity as the EC_50_ of intact extract A (Fig. 2D). Similarly, while extract B had a relative EC_50_ of 20.3 nM (where 1 µg/mL of Mukungulu was calculated to contain 51.0 nM of namushens; Table S2), namushen mixture B had an EC_50_ of only 150 nM, requiring a 7.4-fold higher concentration to achieve similar activity as that observed in intact extract B (Fig. 2E). These results indicate that namushens 1–5, when applied in equal concentrations and proportions to those observed in parental intact Mukungulu extracts, do not fully recapitulate the latency reversal activity of Mukungulu crude extracts on their own.

### Mukungulu robustly reverses HIV-1 latency in PBMC from PLWH stably suppressed on ART

**Fig 3:**
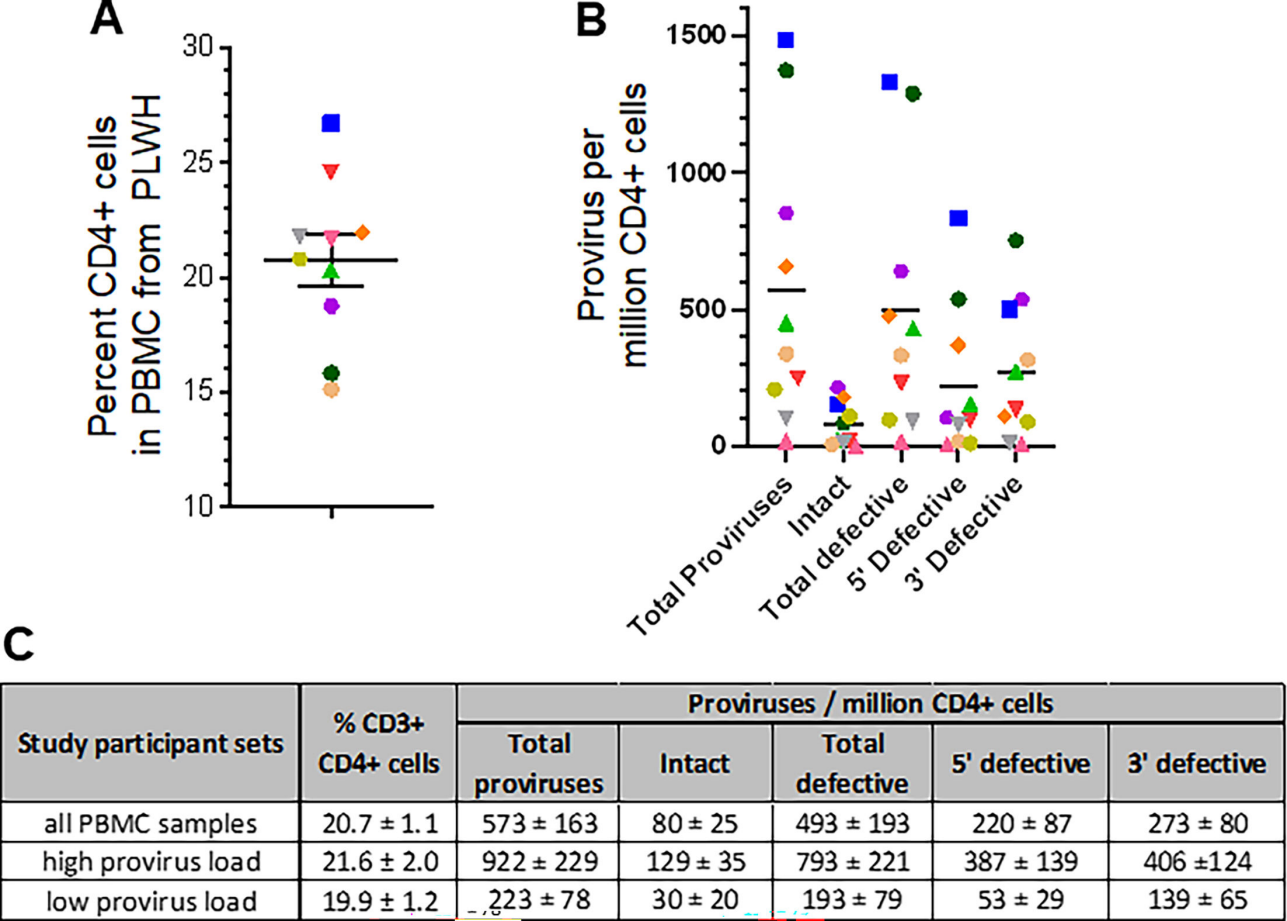
Baseline properties of primary cell samples obtained from 10 ART-suppressed PLWH. (**A**) Percent CD3+ CD4+ T-cells in PBMC. (**B**) Provirus levels in isolated CD4+ T-cells from each donor, as measured by IPDA. In panels **A** and **B**, shapes and colors denote samples obtained from study participants as annotated in Table 1. (**C**) Numerical summaries of data presented in panels **A** and **B**.

To investigate whether latency reversal observed in J-Lat T cell lines extended to primary cells, we obtained PBMC from 10 ART-suppressed PLWH. Table 1 shows the baseline characteristics of study participants. Having first established percent CD4+ T-cells in PBMC from each donor (Fig. 3A), we next quantified levels of intact and defective proviruses within isolated CD4+ T-cells from each study participant using the intact provirus DNA assay (IPDA; Fig. 3B and C) (20). These results indicated an average of 573 ± 163 total proviruses per million CD4+ T-cells, consisting of 80 ± 25 intact proviruses and 493 ± 193 total defective proviruses per million CD4+ T-cells (representing 14.0% ± 4.3% and 86.0% ± 33.7% of total proviruses, respectively).

**TABLE 1:** Baseline characteristics of study participants

Patient ID	Assessed in CD4+ T-cell studies?	Color and shape on graphs	Age	Sex/gender	Race	Ethnicity	Date of HIV diagnosis (mo/yr)	Date of starting ART (mo/yr)	Years of documented continual viral suppression	CD4 nadir recorded at first diagnosis	Current ART regimen	CD4 count on the day of collection	Viral load on the day of collection
1	No	Green-yellow hexagon	45	Male	Not Hispanic	African American	12/1998	2004	11	77	Triumeq	573	<20
2	No	Gray down-triangle	35	Male	Unknown	Unknown	01/2009	06/2010	12	201	Triumeq	478	<20
3	Yes	Green circle	42	Male	Not Hispanic	African American	unk/1998	12/2013	8	Unk, >200	Juluca	826	<20
4	No	Peach circle	43	Male	Not Hispanic	African American	05/2003	2003–2006	12	349	Symtuza	653	51
5	No	Green up-triangle	45	Male	Not Hispanic	African American	01/2011	2011	10	878	Dovato	531	< 20
6	Yes	Orange diamond	59	Male	Not Hispanic	African American	unk/1988	2004	10	Unk, <20	Biktarvy	998	< 20
7	Yes	Red down-triangle	35	Male	Not Hispanic	African American	2008 or 2009	2013–2014	2	Unk	Stribild	942	< 20
8	Yes	Blue square	60	Male	Not Hispanic	White	unk/1998	06/2000	23	Unk, >200	Biktarvy	457	< 20
9	No	Pink up-triangle	51	Male	Not Hispanic	White	12/1997	1997	7	23	Triumeq	712	< 20
10	Yes	Purple hexagon	57	Male	Not Hispanic	African American	01/2002	2004	17	425	Biktarvy	694	< 20

**Fig 4:**
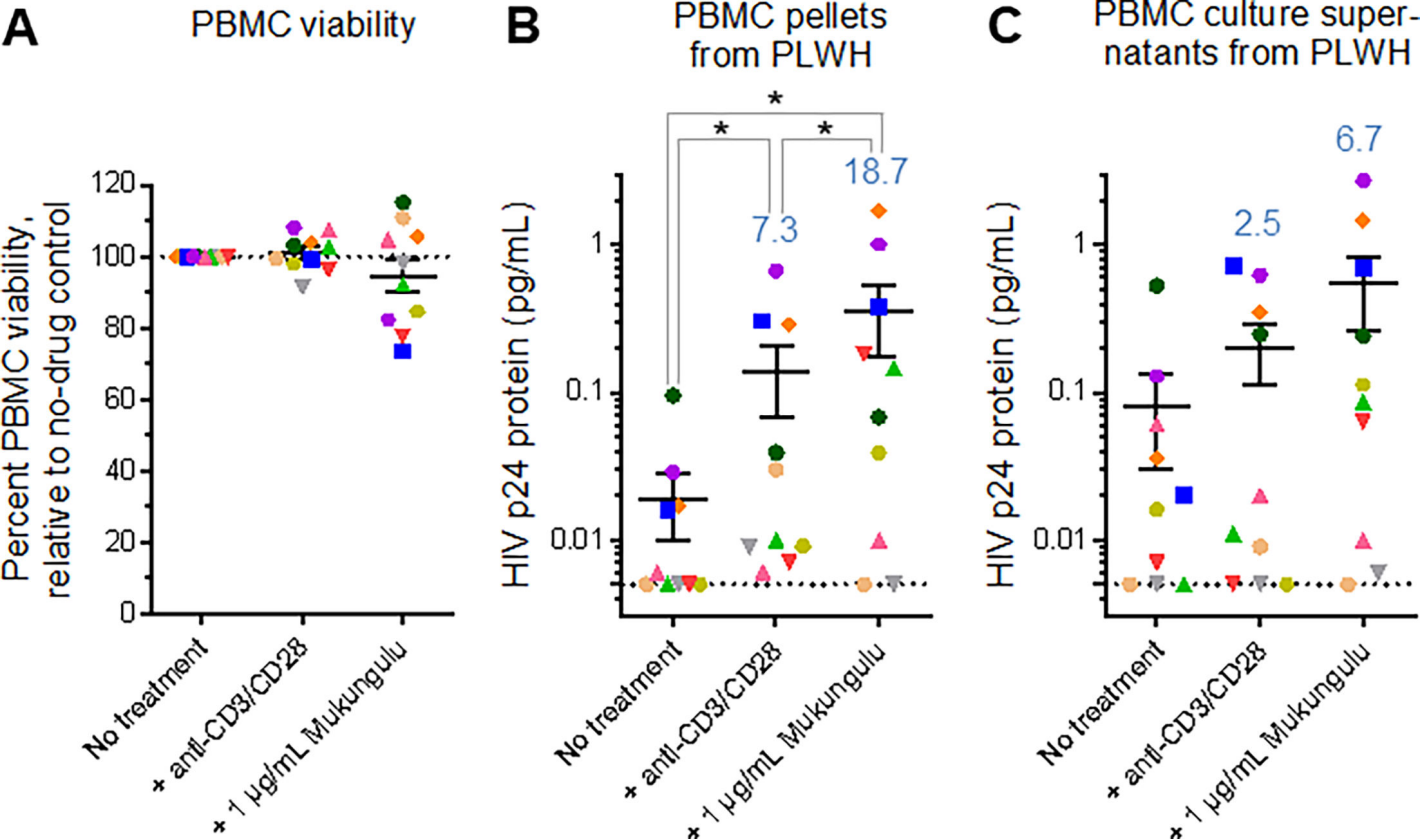
Mukungulu reactivates HIV expression in PBMC obtained from 10 ART-suppressed PLWH. (**A**) Percent cell viability in the presence of LRAs after 72 hours of treatment. Results are presented relative to the viability of untreated cells cultured in parallel. (**B and C**) Detection of gag-p24 protein in cell pellets (**B**) and culture supernatants (**C**) after 72 hours of treatment with LRAs, as measured by Simoa. In all panels, blue values denote average fold increases over no-treatment controls. Shapes located on the dotted line denote gag-p24 protein below the LOD which is annotated here at 0.005 pg/mL. *, *P* < 0.05 as measured by one-sided Mann-Whitney test.

A total of 20 million PBMC from each of the 10 study participants were then cultured in triplicate in the presence of 1 µg/mL of Mukungulu. In these studies, extract B was selected due to a much higher volume of available crude extract. As a positive control, PBMC was also treated in parallel with 50 µg/mL anti-CD3/CD28 dynabeads. After 72 hours of treatment, live cells were quantified by trypan blue stain. As shown in Fig. 4A, we observed donor variance in the degree of viability after anti-CD3/CD28 or Mukungulu treatment, which is likely influenced by cytokine levels and/or proliferation when compared to untreated conditions. However, using this approach, we found that PBMC treated with Mukungulu had 92.4% ± 3.5% viability relative to untreated PBMC (Fig. 4A), indicating no major cytotoxic effects. Cell pellets and culture supernatants were then collected from all experiments and assessed for viral protein production after 72 hours using HIV gag-p24 antigen detection by a single-molecule array (Simoa) (21). Assay reproducibility matched previous results and conservatively defined a lower limit of detection (LOD) of 0.005 pg/mL (21). In untreated cells, limited or no HIV gag-p24 protein was detected across all cell pellets (average 0.019 ± 0.009 pg/mL, assuming a value of 0.005 pg/mL for all samples with gag-p24 below the LOD), while anti-CD3/CD28 induced an average 0.138 ± 0.070 pg/mL of viral protein, or a borderline significant 7.6-fold increase over no-drug control (*P* = 0.05; Fig. 4B). By contrast, Mukungulu treatment induced more gag-p24 production than anti-CD3/CD28 in almost all donors, resulting in an average of 0.354 ± 0.178 pg/mL of viral protein. This increase in viral protein was 2.6-fold more than anti-CD3/CD28 induction (*P* = 0.02) and 18.7-fold more than untreated controls (*P* = 0.02; Fig. 4B), indicating significantly more robust latency reversal by Mukungulu than anti-CD3/CD28 following 72 hours of treatment.

Similar trends were also observed from culture supernatants, although none of these differences were statistically significant. For example, while anti-CD3/CD28 treatment induced an average 0.201 ± 0.089 pg/mL of gag-p24 (a 2.5-fold increase over untreated cells with 0.082 ± 0.052 pg/mL of viral protein; *P* = 0.50), Mukungulu induced an average 0.544 ± 0.285 pg/mL of gag-p24. While this increase by Mukungulu was only 2.7-fold over anti-CD3/CD28 (*P* = 0.19), it produced a 6.7-fold increase over untreated cells (*P* = 0.10; Fig. 4C). These results also support that Mukungulu also induces more latency reversal than anti-CD3/CD28 treatment in PBMC.

### Latency reversal induced by LRAs in PBMC isolated from PLWH correlates with intact HIV provirus levels in CD4+ T-cells

**Fig 5:**
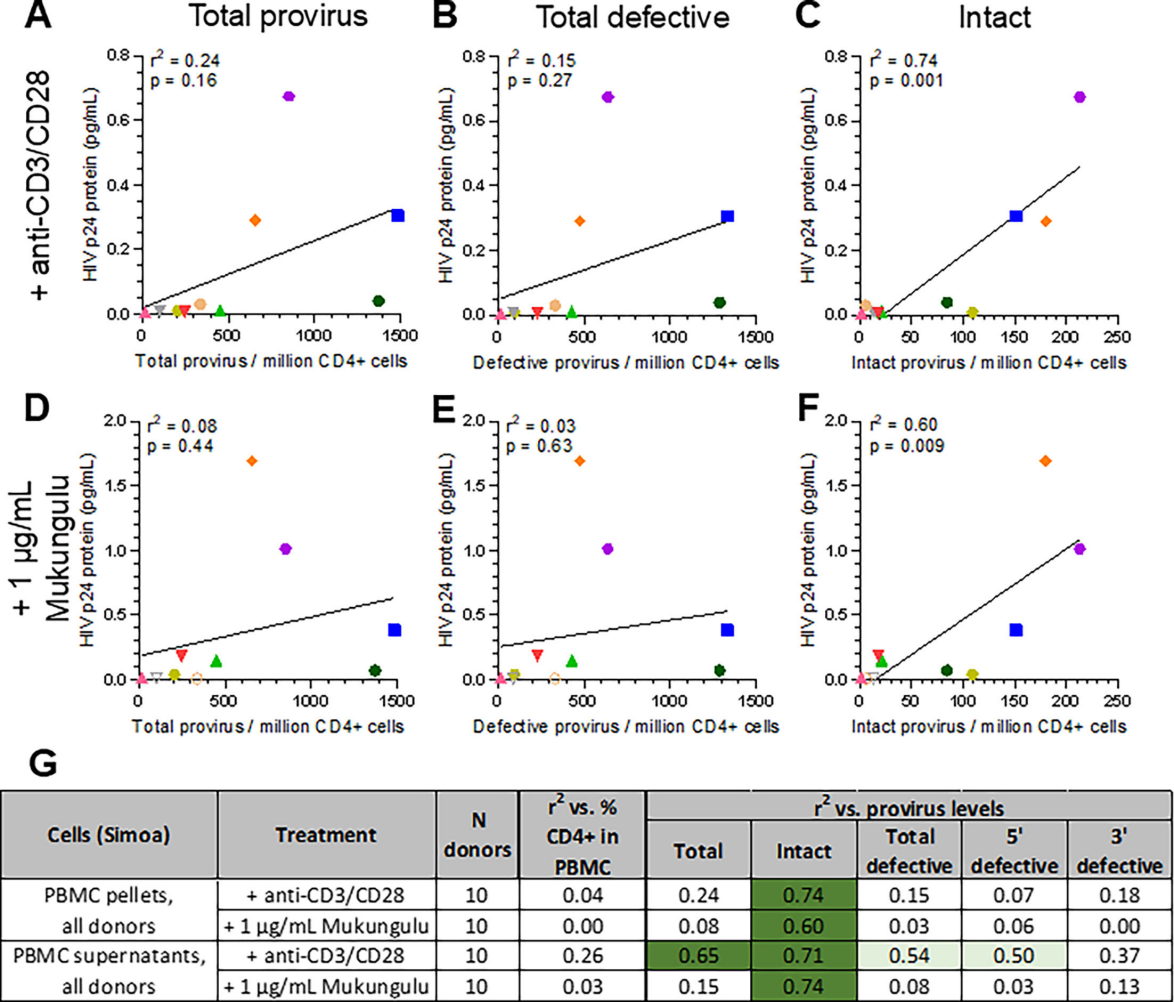
Magnitude of Mukungulu reactivation in PBMC correlates with intact provirus levels in CD4+ T-cells**.** (**A–F**) Correlations of viral protein levels in PBMC pellets following 72 hours treatment with anti-CD3/CD28 (**A–C**) or 1 µg/ml Mukungulu (**D–F**), as measured by Simoa, relative to total provirus (**A and D**), total defective provirus (**B and E**), and intact proviruses (**C and F**) in isolated CD4+ T-cells. In all panels, colors/shapes denote individual donors as annotated in Table 1. (**G**) Correlations of viral protein production relative to provirus levels in isolated CD4+ T cells obtained from 10 PLWH. Unfilled shapes denote gag-p24 protein below the LOD which is annotated here at 0.005 pg/mL. In panel **G**, light green shading denotes *P* < 0.05, and dark green shading denotes *P* < 0.005, as measured by ANOVA.

To explore whether LRA-induced viral protein production correlated with proviral load in CD4+ T-cells, we next compared HIV gag-p24 protein production by stimulated PBMC to provirus levels in isolated CD4+ T-cells as obtained by IPDA. As expected, viral protein expression did not correlate with percent CD3+ CD4+ T-cells, as the cohort had similar frequencies of CD4+ across all study participants (Fig. 3A; Fig. S7). However, we did observe that LRA-induced HIV-1 gag-p24 levels from both PBMC pellets and supernatants from cultured cells treated with anti-CD3/CD28 or Mukungulu correlated significantly with intact but not total or defective provirus levels (Fig. 5; Fig. S7 and S8). For example, while no correlation, as measured by simple linear regression analysis, was observed for viral protein levels in PBMC pellets compared to either total provirus levels in CD4+ T-cells (*r*^2^ = 0.24; *P* = 0.16; Fig. 5A) or total defective provirus levels (*r*^2^ = 0.15; *P* = 0.27; Fig. 5B), a significant correlation was observed when compared to intact provirus levels (*r*^2^ = 0.74; *P* = 0.001; Fig. 5C). A similar pattern was also observed for viral protein from PBMC pellets treated with Mukungulu, with no correlation to total provirus (*r*^2^ = 0.08; *P* = 0.44; Fig. 5D) or total defective provirus (*r*^2^ = 0.03; *P* = 0.63; Fig. 5E) but significant correlation with intact provirus levels (*r*^2^ = 0.60; *P* = 0.009; Fig. 5F). Finally, no correlations were observed for gag-p24 protein measured from PBMC pellets stimulated with anti-CD3/CD28 or Mukungulu when compared to 5′-defective or 3′-defective provirus in CD4+ T-cells (Fig. 5G; Fig. S8).

When culture supernatants were assessed, we similarly observed that HIV gag-p24 protein levels from both anti-CD3/CD28-treated and Mukungulu-treated culture supernatants significantly associated with intact provirus levels in CD4+ T-cells (anti-CD3/CD28 treatment: *r*^2^ = 0.71, *P* = 0.002; Mukungulu treatment: *r*^2^ = 0.74, *P* = 0.001; Fig. 5G; Fig. S9). However, while supernatant gag-p24 induced by Mukungulu did not correlate with total (*r*^2^ = 0.15, *P* = 0.26) or defective provirus levels (*r*^2^ = 0.08, *P* = 0.43), supernatant gag-p24 induced by anti-CD3/CD28 did correlate with both total (*r*^2^ = 0.65, *P* = 0.005) and defective provirus levels (*r*^2^ = 0.54, *P* = 0.02; Fig. 5G; Fig. S9). Similarly, while no correlations were observed for supernatant gag-p24 protein induced by Mukungulu on 5′-defective or 3′-defective provirus in CD4+ T-cells, borderline significance was observed for supernatant gag-p24 protein induced by anti-CD3/CD28 (Fig. 5G; Fig. S9).

Taken together, results support that the magnitude of HIV reactivation induced by both anti-CD3/CD28 and Mukungulu in PBMC *ex vivo* associates primarily with intact provirus levels in CD4+ T-cells.

### Mukungulu reverses HIV-1 latency in CD4+ T-cells isolated from PLWH stably suppressed on ART

To reproduce and extend observations of latency reversal by Mukungulu in ART-suppressed PBMC to the context of CD4+ T-cells, we selected PBMC from study participants in the upper half of HIV provirus load (“high provirus load”), defined as having at least 240 total provirus copies per million CD4+ T-cells. This subset contained an average of 922 ± 229 total proviruses per million CD4+ T-cells, consisting of 129 ± 35 intact proviruses and 793 ± 221 total defective proviruses per million CD4+ T-cells (representing 14.0% ± 3.7% and 86.0% ± 24.0% of total proviruses, respectively; Fig. 3C).

**Fig 6:**
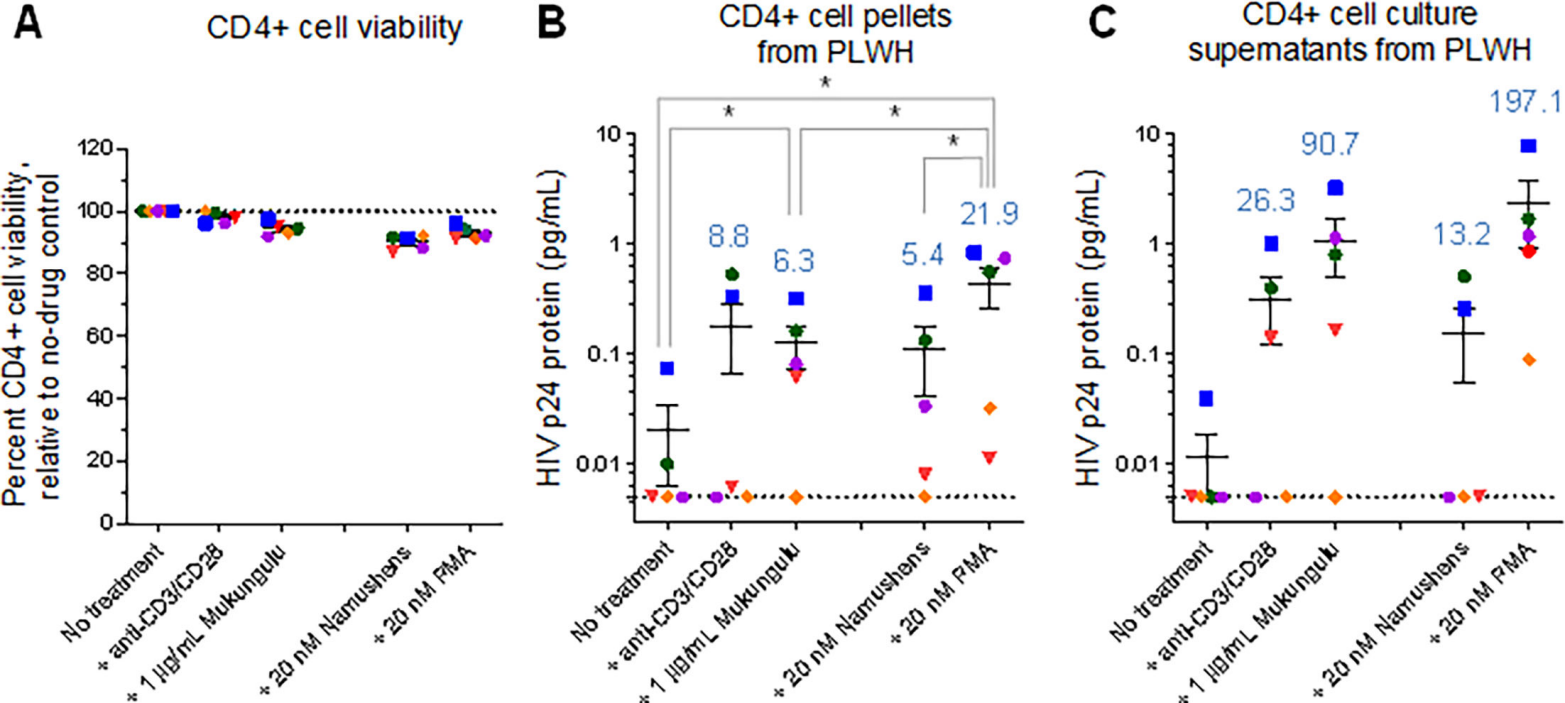
Mukungulu reactivates HIV expression in CD4+ T-cells obtained from five ART-suppressed PLWH. (**A**) Percent cell viability in the presence of LRAs after 72 hours of treatment. Results are presented relative to the viability of untreated cells cultured in parallel. (**B and C**) Detection of gag-p24 protein in cell pellets (**B**) and culture supernatants (**C**) after 72 hours of treatment with LRAs, as measured by Simoa. In all panels, blue values denote average fold increases over no-treatment controls. Shapes located on the dotted line denote gag-p24 protein below the LOD, which is annotated here at 0.005 pg/mL. *, *P* < 0.05 as measured by paired *t*-test.

CD4+ T-cells were next isolated from PBMC from each of the “high provirus load” subset, and 2 million CD4+ T-cells per donor were cultured in duplicate in the presence of 50 µg/mL anti-CD3/CD28 dynabeads or 1 µg/mL Mukungulu (extract B). After 72 hours of treatment, cells in all conditions were again assessed for viability by trypan blue stain, where we found that CD4+ T-cells treated with Mukungulu had 94.4% ± 1.0% viability relative to untreated CD4+ T-cells tested in parallel, again indicating that it did not cause obvious cellular toxicity exceeding cell proliferation *ex vivo* (Fig. 6A). As expected, anti-CD3/CD28 treatment also did not cause obvious toxicity (98.1% ± 0.9% viability relative to untreated cells, respectively; Fig. 6A).

Cell pellets and culture supernatants were again collected from all experiments and assessed by HIV gag-p24 Simoa. In untreated cells, minimal HIV gag-p24 protein was again detected across all CD4+ T-cell pellets (average 0.020 ± 0.014 pg/mL, assuming 0.005 pg/mL gag-p24 protein in samples below the LOD), while anti-CD3/CD28 induced an average 0.175 ± 0.108 pg/mL of viral protein. While this corresponded to an average 8.8-fold increase over untreated cells in viral protein production at 72 hours post-treatment, this increase was not statistically significant (*P* = 0.1; Fig. 6B). While Mukungulu also induced latency reversal in isolated CD4+ T-cells, with an average 0.126 ± 0.055 pg/mL of protein observed over CD4+ T-cells from five PLWH, this corresponded to a significant 6.3-fold increase over untreated cells at 72 hours (*P* = 0.03; Fig. 6B).

When culture supernatants were analyzed, similar trends were again observed, although, like studies from cell pellets, no statistical significance was observed. For example, in untreated cells, we again found minimal gag-p24 protein (average 0.020 ± 0.014 pg/mL). Anti-CD3/CD28 treatment induced an average 0.311 ± 0.189 pg/mL of viral protein in supernatants, again corresponding to a borderline significant 26.3-fold increase over untreated cells (*P* = 0.09; Fig. 6C). However, Mukungulu induced an average 1.075 ± 0.585 pg/mL of viral protein, which corresponded to a 3.5-fold increase over anti-CD3/CD28-treated cells (*P* = 0.07) but a 90.7-fold increase over untreated cells (*P* = 0.07).

To confirm whether the activity differences between Mukungulu and namushen mixtures (Fig. 2C and D) extended to *ex vivo* observations, we also treated in parallel CD4+ T-cells from the same five donors with 20 nM namushens (i.e., corresponding to concentration present in 1 µg/mL of Mukungulu extract B; Table S2). As positive controls, isolated CD4+ T-cells were also treated with 20 nM PMA, which represented maximal stimulation of J-Lat cells (as shown in Fig. 2C and D). Following trypan blue stain, we found that CD4+ T-cells treated with namushens had 90.2% ± 1.1% viability relative to untreated CD4+ T-cells, while cells treated with PMA had 94.4% ± 1.0% viability (Fig. 6A). However, similar to *in vitro* observations, treatment with the corresponding namushen mixture on CD4+ T-cells showed only a borderline significant increase in gag-p24 production in both cell pellets (5.4-fold; *P* = 0.09; Fig. 6B) and culture supernatants (13.2-fold; *P* = 0.11; Fig. 6C), in both cases not achieving the latency reversal observed by Mukungulu. By contrast, PMA achieved a much larger induction in both cell pellets and culture supernatants (21.9- and 197.1-fold; *P* = 0.03 and 0.09, respectively; Fig. 6B and C).

Taken together, results demonstrate that Mukungulu is a robust latency-reversing agent with direct activity on CD4+ T-cells as well as activity that is comparable to or exceeding that of anti-CD3/CD28 stimulation after 72 hours, particularly in culture supernatants. These data also support that an isolated namushen mixture does not match the viral antigen reactivation observed with Mukungulu, further indicating other factors in Mukungulu that may support HIV reactivation and replication over namushen-only based strategies.

### Mukungulu extract is tolerated and reverses latency *in vivo*

To determine whether the amount of phorbol esters in Mukungulu would be tolerated by mice, we first injected a total of 18 CD-1 mice intraperitoneally with Mukungulu at 0.125, 1.25, or 12.5 mg/kg (three female + three male mice per concentration) and monitored them for tolerance up to 72 hours post-administration. No changes in behavior were observed for any mouse at 0.125 mg/kg administration other than ruffled fur and slightly reduced activity at 4 hours post-administration. At higher concentrations, mice began to exhibit lethargy, hunched postures, slightly labored breathing, ruffled fur, and/or slightly reduced activity starting at 10 minutes post-administration, which resolved after 8 hours other than slight piloerection (Table S3). No other changes were observed, including changes in body weight up to 72 hours post-administration, indicating that mice tolerated Mukungulu treatment at concentrations up to 12.5 mg/kg, albeit with temporary side effects (Table S3).

**Fig 7:**
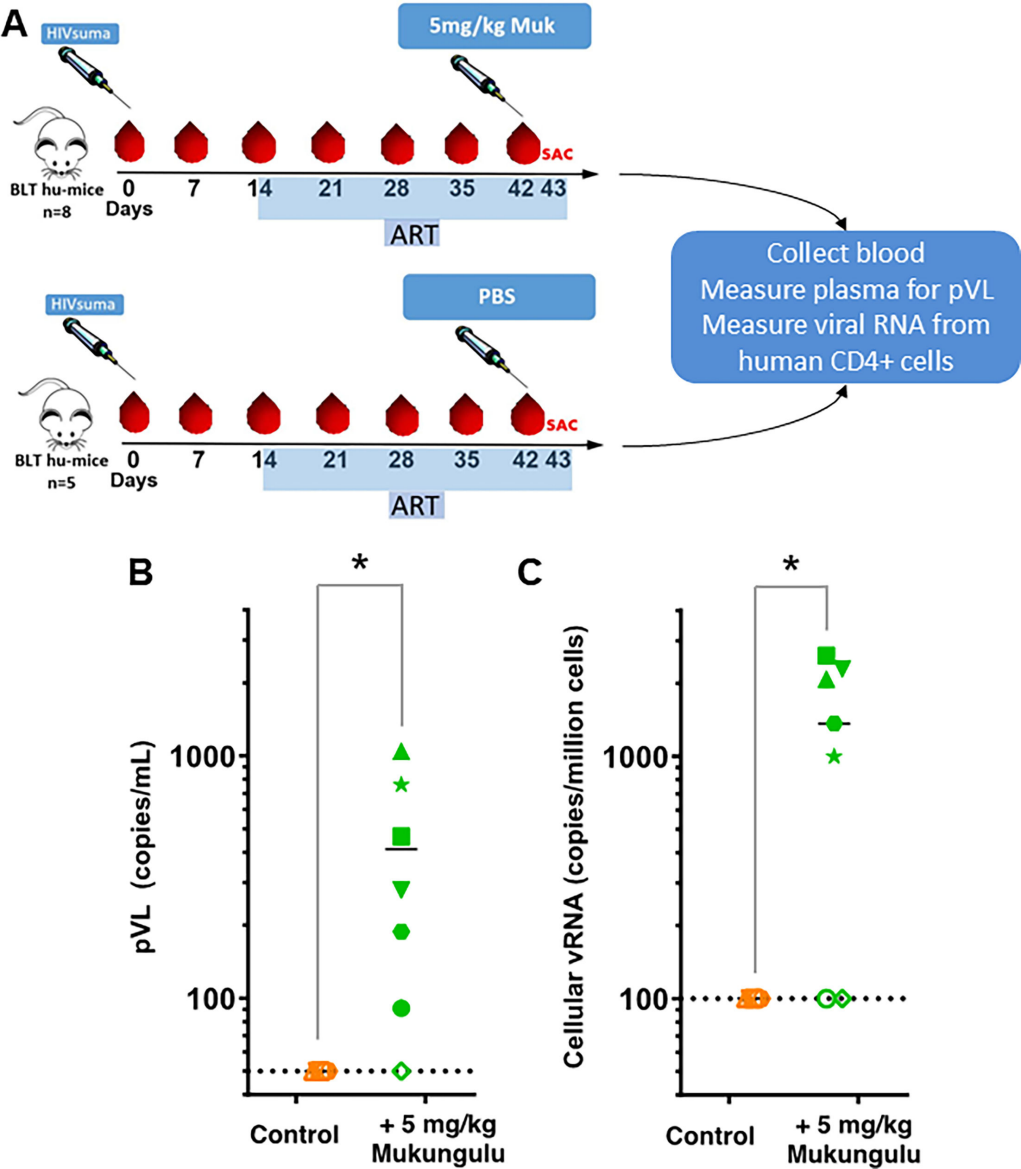
Mukungulu reverses HIV-1 latency *in vivo.* (**A**) Schematic illustration of the BLT humanized mice model-based study. (**B**) pVL rebound measurements in ART-suppressed BLT humanized mice, treated with Mukungulu. (**C**) Viral RNA induction from isolated human CD4+ T-cells from ART-suppressed mice treated with PBS vehicle control or Mukungulu. Colors/shapes denote individual mice. *, *P* < 0.05 as measured by the one-sided Mann-Whitney test.

We next investigated whether the latency reversal induced by Mukungulu *ex vivo* corresponded to *in vivo* efficacy using the ART-suppressed HIV-infected bone marrow-liver-thymus (BLT) humanized mice model (22–25). Here, 12 HIV-infected, ART-suppressed BLT humanized mice were placed into two experimental groups per the schematic study design shown in Fig. 7A. Following immune reconstitution, HIV infection, and detection of plasma viral load after 2 weeks, ART was initiated for an additional 4 weeks. Viral suppression in plasma was observed for 10 out of 12 mice after 3 weeks of ART and for all 12 mice after 4 weeks (Table S4). The test group then comprised seven mice receiving 5 mg/kg Mukungulu extract (extract B), while the remaining five received phosphate-buffered saline (PBS) vehicle control (Fig. 7A). After 24 hours, mice were euthanized, and blood was collected to measure pVL and cellular viral RNA from isolated human CD4+ T-cells. Unlike CD-1 mice, BLT humanized mice treated with 5 mg/kg Mukungulu exhibited weight loss after 24 hours, where body weight was 93.5% ± 7.7% of weight before treatment, in contrast to PBS-treated mice where body weight was 99.8% ± 1.3% of pre-treatment (*P* = 0.05). This indicated poorer tolerance of Mukungulu by HIV-infected BLT humanized mice when compared to wild-type mice, which likely reflects experimental and/or strain-specific features. However, as shown in Fig. 7B, virus expression was readily detected following Mukungulu treatment, where an average pVL of 550 ± 130 viral copies/mL was observed, compared to the control cohort where the plasma viral load remained below the limit of detection (*P* = 0.009). Furthermore, and consistent with the plasma viral load rebound results, cellular viral RNA expression in isolated human CD4+ T-cells was observed in Mukungulu-treated mice averaging 1870 ± 250 viral mRNA copies per million cells compared to no detection in those animals that received vehicle control PBS (*P* = 0.03; Fig. 7C). Taken together, these results indicate that Mukungulu is a robust LRA at concentrations that can be tolerated *in vivo*.

## DISCUSSION

Having previously documented the traditional use of “Mukungulu,” a bark extract of *Croton megalobotrys*, to manage HIV/AIDS to supplement ART in northern Botswana (16, 17), here, we show that Mukungulu can act as a robust LRA *ex vivo* in both PBMC and isolated CD4+ T-cells from PLWH as well as *in vivo* using a humanized mouse model. Mukungulu extract is also shown to be tolerated in uninfected control mice at doses able to induce latency reversal by an active component of Mukungulu-specific phorbol esters (namushens 1–5). Notably, the tolerability of Mukungulu stands in direct contrast to the established *in vivo* toxicities of PMA and other phorbol esters (12, 13). Furthermore, as Mukungulu’s traditional use as a single dose correlates with documented improvement in patient health (16, 17), its activity as an LRA described here raises the hypothesis that it may also be tolerated if used as an LRA strategy in conjunction with ART. Additional support for the use of PKC activators in reactivating HIV comes from the use of *Euphorbia kansui* herbs in Chinese traditional medicine, which, although not in use for HIV/AIDS management, does contain PKC-activating ingenols with LRA activity (26, 27).

Extensive bioassay-guided fractionation of 81.3 g of Mukungulu crude extract identified a total of five pure namushen phorbol esters. These namushens comprise 1.2%–3.1% of the total phytochemical composition of Mukungulu and were present, at least to a first approximation, in similar proportions across two independent collections of Mukungulu extracts. However, differences in namushen levels were observed between these extracts, indicating variability that arises either naturally, during traditional collection, and/or during extract preparation. As a result, further development of a Mukungulu as an LRA therapy will require standardizing and identifying optimal concentrations of namushens in future Mukungulu preparations.

Additionally, when namushen mixtures were reconstituted to the concentrations and proportions seen in parental extracts, they did not recapitulate the full latency-reversing profiles associated with their respective crude extracts in both J-Lat cells and culture supernatants from isolated CD4+ T-cells from ART-suppressed PLWH. However, when the DMSO stocks of Mukungulu extract B were frozen and thawed 10 times, the proportions of namushens were essentially identical, indicating *in vitro* stability. Therefore, results suggest that isolated namushen mixtures reverse latency more slowly and/or that parental Mukungulu extracts more effectively induce viral expression after reactivation. While bioassay-guided fractionation indicates that isolated namushens are likely the dominant LRA species in Mukungulu, our results do not exclude the presence of additional, unidentified phytoconstituents in Mukungulu that could enhance namushen-driven latency reversal. In support of this hypothesis, minor LRA species which can enhance the activity of known LRAs have been recently reported from other related plant species (28, 29).

Reversal of HIV latency by Mukungulu was also observed in both PBMC and isolated CD4+ T-cells from up to 10 ART-suppressed PLWH. Notably, the activity of 1 µg/mL Mukungulu was generally on par with, or superior to, anti-CD3/CD28 polyclonal T-cell positive control activity. As expected, the higher the intact proviral load of HIV in CD4 T-cells, the better the detection of reactivation by LRAs *ex vivo*. We also observed that Mukungulu’s activity in isolated CD4+ T-cell pellets, where gag-p24 increased 6.3-fold relative to untreated cells, was lower than results from PBMC pellets, where Mukungulu induced an 18.7-fold increase, suggesting that Mukungulu may amplify its effects over 72 hours with other contributing cell types apart from direct action on CD4+ T-cells. Effects of Mukungulu on other cell types within PBMC like CD8+ T-cells remain to be explored. We also saw a significant increase in gag-p24 protein induced by Mukungulu in the pellets of both PBMC and CD4+ T-cells, although these trends remained borderline significant in supernatants. As the levels of gag-p24 protein detected across donors here resemble results from previous studies (21), it is likely that a sufficient number of PBMC (20 million) or CD4+ T-cells (2 million) per condition was used to detect even low-level latency reversal by LRAs. As a result, the preponderance of borderline significant comparisons, particularly in studies of culture supernatants, likely reflects both defining a conservative LOD in *ex vivo* studies, expected donor-to-donor variability, and limited ability to reverse latency from cells from a subset of donors (21). It could also indicate a lack of viral maturation and/or gag-p24 release, which limits viral protein production within cells.

Importantly, and in further support of these *ex vivo* studies, we used ART-suppressed humanized BLT mice infected with HIV (22–25) to demonstrate that a single 5 mg/kg injection of Mukungulu robustly reactivates HIV expression in most animals after 24 hours, as measured by both pVL and cell-associated HIV RNA levels. The magnitude of latency reversal induced by 5 mg/kg Mukungulu after 24 hours approximates what is reported for plasma viral RNA reactivation in a similar ART-suppressed BLT humanized, HIV-infected mouse model treated with 3 mg/kg of the SMAC mimetic AZD5582 after 48 hours (30), further supporting robust Mukungulu activity *in vivo*. Mice received a single dose of Mukungulu as this mirrors the traditional use of Mukungulu, which is taken once followed by healer observation for 48–72 hours (16, 17). It should be stressed, however, that HIV-infected humanized mice did not tolerate Mukungulu as well as control wild-type CD-1 mice, as measured by weight loss. We interpret this to be due to humanized mice being more sensitive to activation as created from immunodeficient mice known to be less robust than wild-type mice. Future studies will be needed to identify the presence of any additional toxicity outcomes in cells and/or tissues associated with Mukungulu use in BLT vs wild-type mice. Similar weight loss was also observed in postnatal Wistar rats following treatment with 10-fold lower concentrations of PMA at 500 µg/kg (13). It is also of interest to note our previous field documentation where we learned that traditional health practitioners do not administer Mukungulu to persons who appear excessively weak (17). Future investigation in both murine and nonhuman primate models will need to better define *in vivo* toxicity thresholds as well as changes in proinflammatory cytokine responses associated with *in vivo* Mukungulu dosing. It will also be important to monitor the pharmacokinetics of namushens and any changes to the viral reservoir in subsequent *in vivo* studies.

One important limitation of these studies is that all study participants were male and originated from the northeast United States, a predominant region for HIV subtype B. As subtype C is predominant in Botswana and Southern Africa, *ex vivo* studies need to be extended to specimens from PLWH in these areas, particularly given recent reports that LRAs may have more activity against latent subtype C proviruses relative to subtype B (31). Another limitation is the difference in how Mukungulu was administered in mice when compared to traditional use in humans. Specifically, mice received an intraperitoneal (i.p.) injection of crude Mukungulu extract, while humans are documented to ingest one leveled teaspoon of Mukungulu powder prepared in a small teacup filled with boiling hot porridge that is mixed until cold (17). Future studies will need to further investigate formulation strategies for Mukungulu administration relative to HIV reactivation activity. While challenging to execute, another possibility is to observe the traditional use of Mukungulu by PLWH while monitoring for changes in plasma viral loads and/or provirus levels in CD4+ T-cells.

In summary, we show that Mukungulu is a robust candidate LRA in primary cells from PLWH and in HIV humanized mouse models where reactivation activity doses were also documented to be tolerated despite the presence of PKC-activating phorbol esters. The identification of Mukungulu as an LRA strategy as a consequence of reverse pharmacology in collaboration with indigenous traditional medicine also stresses that current uses of medicinal plants globally may hold information on tolerability and impact on HIV disease yet to be identified.

## MATERIALS AND METHODS

### Cells, viruses, animals, reagents, and biosafety

J-Lat T cell clones 9.2 and 10.6 were obtained from the NIH AIDS Reagent Program, Division of AIDS, NIAID, and NIH (contributed by Dr. Eric Verdin) (19). Cells were cultured in R10+ medium (RPMI 1640) with HEPES and L-glutamine, 10% fetal bovine serum, 100 units/mL of penicillin, and 100 µg/mL streptomycin.

PBMCs and CD4+ T cells were isolated from whole blood obtained from 10 ART-suppressed PLWH enrolled in the BEAT-HIV Collaboratory study (Table 1) by way of written informed consent. At the time of blood sample collection, study participants had plasma HIV RNA levels below the limit of detection (<20 copies/mL plasma viral load; only one donor had 51 copies/mL pVL).

All test agents were diluted in DMSO and used immediately or stored frozen at −20°C until use. All primary cell and animal studies, including infectious HIV, were conducted under Biosafety Level 2+ laboratory conditions.

### Collection of Mukungulu

*Croton megalobotrys* Müll Arg. (“Mukungulu”) bark was collected by the traditional healer S. Simonambango in 2018 around Maun, Ngamiland District, North-Western Botswana. Four hundred and eighty gram (480 g) of the bark was ground and extracted with CH_2_Cl_2_/methanol as previously described (17), giving rise to 102.8 g of an oily dark brown extract (yield: 21.4%). A total of 81.3 g of this extract served as the starting material for the bioassay-guided fractionation reported here (extract A). Extract B represented the methanolic extract from original *Croton megalobotrys* bark collected by the healer S. Simonambang in 2014 in the Kazungula District covering the Zambia/Botswana border region, which was botanically authenticated as previously described (voucher specimen KM-Ks-3-2015) (17)

### *In vitro* HIV-1 latency reversal model

HIV-1 latency reversal profiles of test agents (Mukungulu Extracts and its novel derivatives) were measured as previously described using flow cytometry-based GFP reporter cells (J-Lat 10.6 cells) that contain an integrated HIV-1 provirus, modeling HIV-1 latency *in vitro* (19). Basically, J-Lat 10.6 cells were plated in 96-well plates (i.e., 2 × 10^5^ cells per well at a final volume of 200 µL) and treated with multiple concentrations of test agents, alongside an established positive control LRA, and PMA and incubated at 37°C and 5% CO_2_ for 24 hours. Cells were then assessed and detected for GFP expression usin flow cytometry, where GFP-positive cells indicated latent HIV-1 reactivation. DMSO (0.1%) was used as a vehicle control.

### Bioassay-guided fractionation of Mukungulu extracts

Latency reversal properties of Mukungulu extract fractions were determined as described above using J-Lat 9.2 cells. Details on the isolation and structural characterization of namushens 1–5 can be found in the Supplemental Material.

### Namushen quantitation in Mukungulu extracts

Mukungulu extracts were each diluted 20-, 40-, and 80-fold with 80% methanol. Diluted samples were analyzed by LC-MS using a Thermo Scientific Q Exactive HF-X mass spectrometer in-line with a Thermo Scientific Vanquish UHPLC system and reversed-phase Synergi Polar-RP C18 column (Phenomenex). Replicate injections were performed for each sample. Namushens were detected as formate adducts in negative ion mode, similar to published results for related phorbol esters (32). The concentrations of namushens were determined from MS peak integrations using calibration curves generated from purified compounds diluted into 80% methanol (0.005–10 µM range, quadratic fit, 1/×2 weighting). Quantitation was highly reproducible with most namushens in the Mukungulu extracts having coefficients of variation of less than 10% among the replicate injections and across different dilutions.

### Percent CD3+ CD4+ T-cell detection in PBMCs

Freshly thawed PBMC were resuspended to 10^6^ cells/mL in PBS and stained with 1 µL of Aqua LIVE/DEAD stain (Thermo Fisher) and incubated in the dark for 30 minutes. Cells were then washed, resuspended in PBS to 3 × 10^6^ cells/mL in PBS, distributed into 100 µL aliquots, and stained with 5 µL each of antibody CD4-V450 (clone: RPA-T4; BD Biosciences) and CD3-PE-CF594 (clone: UCHT1; BD Biosciences). After 15 minutes of incubation in the dark, cells were treated with 3 mL 1× BD FACs Lysing Solution (BD Biosciences) for 10 minutes in the dark, washed with FACs Wash Buffer (BD Biosciences), and resuspended in 200 µL FACs Wash Buffer. A total of 100,000 events were then collected on a BD Biosciences LSRII flow cytometer. Flow cytometry data were analyzed using FlowJo v. 10.10.0 software (FlowJo LLC, Ashland, OR, USA).

### IPDA quantification of persistent HIV-1 proviral DNA

In this study, we employed the IPDA method to quantify persistent intact and defective HIV-1 proviral DNA in CD4+ T-cells isolated from 10 study participants. An in-depth description of the IPDA design rationale is available in Bruner et al. (20) Sample processing and IPDA measurements were performed by Accelevir Diagnostics under company standard operating procedures by blinded operators. Briefly, cryopreserved PBMCs were viably thawed, and total CD4+ T-cells were obtained via negative immunomagnetic selection (EasySep Human CD4+ T-cell Enrichment Kit, Stemcell Technologies), with cell count, viability, and purity assessed by flow cytometry both before and after selection. Genomic DNA was isolated using the QIAamp DNA Mini Kit (Qiagen) with RNA removed by RNase A treatment. DNA concentrations were determined by fluorometry (Qubit dsDNA BR Assay Kit, Thermo Fisher Scientific), and DNA quality was determined by UV-visible spectrophotometry (QIAxpert, Qiagen). Genomic DNA was then analyzed by IPDA using the Bio-Rad QX200 AutoDG Droplet Digital PCR system, and results were reported as frequencies of intact, defective, and total proviruses per million input cells.

### HIV-1 gag-p24 single-molecule array

CD4+ T-cells were isolated from frozen PBMCs from five study participants using the EasySep Human CD4+ T-cell Enrichment Kit (Stemcell Technologies). A total of 2 million CD4+ T-cells were then cultured in 96-well plates in duplicate in 200 µL R10+ media supplemented with 100 U/mL IL-2, 200 nM raltegravir, and appropriate test agents (e.g., 50 µg/mL anti-CD3/CD28 dynabeads [Thermo Fisher] according to manufacturer instructions, Mukungulu extract, Namushen mixtures, PMA, or 0.1% DMSO vehicle control). For PBMC-based studies, 20 million cells were cultured in 12-well plates in triplicate in 2 mL of R10+ media plus 100 U/mL IL-2, 200 nM raltegravir, and test agents. Cells were then incubated for 72 hours at 37°C and 5% CO_2_. Following incubation, live cells were quantified by trypan blue stain, and cell pellets and culture supernatants were harvested for HIV gag-p24 protein Simoa. Cell pellets were resuspended in Simoa buffer (49% blocker casein in PBS [Thermo-Fisher], 49% fetal bovine serum [FBS], 1% triton, and 1× protease inhibitor cocktail) and incubated for 30 minutes, while culture supernatants were mixed with 10% triton (in PBS) to a final concentration of 1%, before storage at −80°C.

Samples were then analyzed for gag-p24 protein using Simoa methods described previously by Wu et al. (21). For detection of viral protein, anti-p24 conjugated magnetic beads were added to thawed cell pellet lysate in Simoa buffer and supernatant samples to enrich for gag-p24. Bound gag-p24 was eluted with 100 µL 0.1% trifluoroacetic acid, and the eluate was neutralized with 20 µL of 1 M Tris-HCl at pH 9.0. Meanwhile, the beads were eluted with 100 µL of 3% bovine serum albumin (BSA) in PBS again to obtain residual p24. Both eluates were combined into a 200 µL final volume. A total of 140 µL of each sample was then analyzed on a Quanterix HDX-1 platform using gag-p24 Simoa kits obtained from Quanterix. Gag-p24 concentrations were calculated with Simoa software that uses four-parameter logistic regression curve fitting and reported as picograms per milliliter of cell lysate or culture supernatant.

### *In vivo* tolerability of Mukungulu

Tolerability studies were performed at Alliance Pharma (Malvern, PA, USA). Eighteen mice (nine female + nine male) were injected intraperitoneally with 0.125, 1.25, or 12.5 mg/kg of Mukungulu crude extract (*n* = 6 each) and evaluated changes in behavior for the first 30 minutes post-administration, every 4–6 hours for the first 24 hours, and then every 12–24 hours up to 72 hours.

### Generation of BLT humanized mice

Two independent cohorts of BLT mice were generated as previously described in accordance with the Wistar Institute Animal Care and Research Committee regulations (protocol 201360) (22–25). Briefly, female NSG mice aged 6–8 weeks (NOD.Cg-Prkdcscid Il2rgtm1Wjl/SzJ, Jackson Laboratory) were pretreated with busulfan at 30 mg/kg and implanted with human fetal thymic tissue fragments and fetal liver tissue fragments under the murine renal capsule. Following surgery, mice were injected via the tail vein with CD34+ hematopoietic stem cells. Human fetal liver and thymus tissues were procured from Advanced Bioscience Resources (Alameda, CA). Twelve weeks post-surgery, human immune cell reconstitution in peripheral blood was determined by flow cytometry.

### HIV infection, antiretroviral therapy suppression, and Mukungulu treatment

BLT mice from each cohort were randomly divided into two groups and infected intravenously with 104 × 50% tissue culture infectious dose of HIV_SUMA_ in Biosafety Level 2+ conditions. Peripheral blood was collected weekly for plasma viral load assays. Two weeks after infection, mice were placed on a diet combined with ART (1,500 mg/kg emtricitabine, 1,560 mg/kg tenofovir-disoproxil-fumarate, and 600 mg/kg raltegravir). Four weeks post-ART, mice were treated with PBS control or 5 mg/kg Mukungulu by intraperitoneal injection. After 24 hours, mice were euthanized, and blood was collected. Plasma viral loads were measured as previously described (22–25). Single-cell suspensions were generated using the gentleMACS Octo Dissociator (San Diego, CA). RNA was extracted using AllPrep DNA/RNA/miRNA Universal Kit (Qiagen, catalog # 80224). Cell-associated HIV RNA was measured as previously described (22–25).

### Data and statistical analyses

All results were analyzed using Graphpad Prism v. 10.2.0 (Boston, MA, USA). For *in vitro* studies, results denote the mean ± SEM from at least three independent experiments. For *ex vivo* studies, each data point denotes average results from a given study participant sample performed in duplicate (for CD4+ T-cells) or triplicate (for PBMC). For *in vivo* studies, each data point denotes average results from a given animal performed in duplicate. Statistical significance was determined using either a paired *t*-test, one-sided Mann-Whitney test, or ANOVA, where a *P*-value < 0.05 was considered significant.

## Data Availability

All data are available in the main text or the supplemental material.

## SUPPLEMENTAL MATERIAL

Supplemental material mbio.03816-24-s0001.docx Supplemental text, Tables S1–S4, and Figures S1–S9.

ASM does not own the copyrights to Supplemental Material that may be linked to, or accessed through, an article. The authors have granted ASM a non-exclusive, world-wide license to publish the Supplemental Material files. Please contact the corresponding author directly for reuse.
