# Supplementary material for: Ex vivo and in vivo HIV-1 latency reversal by “Mukungulu,” a protein kinase C-activating African medicinal plant extract
Source: mBio. 2025 Apr 23;16(5):e03816-24. doi: 10.1128/mbio.03816-24 (PMC12077168; doi:10.1128/mbio.03816-24)
Supplement: Supplemental material — Supplemental text, Tables S1–S4, and Figures S1–S9. [file mbio.03816-24-s0001.docx]

Supplementary Materials for

**Ex vivo and in vivo HIV-1 latency reversal by “Mukungulu,” a protein kinase C-activating African medicinal plant extract**

Khumoekae Richard *et al.*

*Corresponding author. Email: [itietjen@wistar.org](mailto:itietjen@wistar.org); [montaner@wistar.org](mailto:montaner@wistar.org)

**This PDF file includes:**

Supplementary Text

Tables S1 to S4

Figs. S1 to S9

Supplementary Text

Isolation and identification of novel compounds from the Mukungulu plant crude extract.

General experimental procedures, collection and extraction of plant material and isolation of namushens 1-5 were as described previously for the isolation of namushens 1 and 2.^15^

Namushen-3 was isolated as a clear glass [UV (4:1 MeCN/H_2_O) λ_max_ 207, 253 nm; ^1^H and ^13^C NMR see **Table S1**; positive ion HRESITOFMS [M + Na]+ *m/z* 623.3554 (calculated for C_35_H_52_O_8_Na, 623.3554), appropriate for a molecular formula of C_35_H_52_O_8_, requiring 10 sites of unsaturation, one more than that observed in namushens-1 and 2]. Comparison of the ^1^H/^13^C/gCOSY/gHSQC/gHMBC/tROESY NMR data of namushen-3 (**Table S1**, **Figures S1-S2**) with that of namushens-1 and 2 along with the additional site of unsaturation revealed that in namushen-3 the 2-methylbutanoate ester residue at C-17 in namushen-1 had been replaced by the olefin containing tiglic acid residue and that namushen-3 could be assigned the structure drawn in **Figures S1-S2**.

Namushen-4 was isolated as a clear glass [UV (9:1 MeCN/H_2_O) λ_max_ 201, 249 nm; ^1^H and ^13^C NMR see **Table S1**; positive ion HRESITOFMS [M + Na]+ *m/z* 651.3884 (calculated for C_37_H_56_O_8_Na, 651.3867), appropriate for a molecular formula of C_37_H_56_O_8_, requiring 10 sites of unsaturation, the same as namushen-3]. The NMR spectra of namushen-3 and namushen-4 were remarkably similar (**Table S1**, **Figures S3-S4**), and the structures of the two compounds were found to only differ in the length of the alkyl chain of the lipid of the ester functionality at C-13. The structure of namushen-4 was assigned as drawn in **Figures S3-S4**.

Namushen-5 was isolated as a clear glass [UV (9:1 MeCN/H_2_O) λ_max_ 200, 235 nm; ^1^H and ^13^C NMR see **Table S1**; positive ion HRESITOFMS [M + Na]+ *m/z* 525.3190 (calculated for C_30_H_46_O_6_Na, 525.3187), appropriate for a molecular formula of C_30_H_46_O_6_, requiring 8 sites of unsaturation]. Comparison of the NMR spectra of namushen-5 (**Table S1**, **Figures S5-S6**) to that of namushens-3 and 4 revealed that namushen-5 lacked substitution at C-17 and was assigned the structure drawn in **Figures S5-S6**.

**Table S1.** ^13^C and ^1^H NMR Data for namushens 3-5 and Comparison with namushen-1 Recorded in C_6_D_6_.

|  | **Namushen-3** | | **Namushen-4** | | **Namushen-5** | | **Namushen-1** | |
| --- | --- | --- | --- | --- | --- | --- | --- | --- |
| Position # | *δ_C_* | *δ_H_* (*J* in Hz) | *δ_C_* | *δ_H_* (*J* in Hz) | *δ_C_* | *δ_H_* (*J* in Hz) | *δ_C_* | *δ_H_* (*J* in Hz) |
| 1 | 160.4 | 7.42 (bs) | 160.6 | 7.43 (bs) | 160.9 | 7.48 (bs) | 160.4 | 7.42 (bs) |
| 2 | 133.0 | / | 133.0 | / | 132.9 | / | 132.9 | / |
| 3 | 208.1 | / | 208.3 | / | 208.7 | / | 208.1 | / |
| 4 | 73.8 | / | 73.8 | / | 74.0 | / | 73.8 | / |
| 5 | 38.8 | 2.23 (bd, 18.9)  2.36 (bd, 18.9) | 38.8 | 2.30 (bd, 18.9)  2.39 (bd, 18.9) | 38.6 | 2.43 (bd, 19.1)  2.48 (bd, 19.1) | 38.8 | 2.25 (bd, 18.9)  2.37 (bd, 18.9) |
| 6 | 140.6 | / | 140.7 | / | 140.0 | / | 140.6 | / |
| 7 | 129.0 | 5.61 (bd, 5.2) | 129.1 | 5.63 (bd, 5.0) | 129.3 | 5.71 (bd, 5.1) | 129.0 | 5.60 (bd, 5.4) |
| 8 | 38.9 | 3.13 (bt, 5.2) | 38.9 | 3.16 (bt, 5.0) | 38.9 | 3.17 (bt, 5.1) | 38.9 | 3.11 (bt, 5.4) |
| 9 | 76.1 | / | 76.2 | / | 75.6 | / | 76.1 | / |
| 10 | 56.2 | 3.43 (bs) | 56.2 | 3.45 (bs) | 55.6 | 3.48 (bm) | 56.2 | 3.43 (bs) |
| 11 | 36.6 | 2.05^b^ | 36.6 | 2.08^b^ | 36.1 | 2.13 (m) | 36.6 | 2.05^b^ |
| 12 | 32.5 | 1.79 (m)  2.07^b^ | 32.5 | 1.79 (dd, 16.7, 13.7)  2.08^b^ | 31.9 | 1.79 (dd, 14.2, 11.3)  2.08^b^ | 32.4 | 1.78 (dd, 16.5, 13.7)  2.05^b^ |
| 13 | 63.5 | / | 63.5 | / | 63.1 | / | 63.5 | / |
| 14 | 31.5 | 1.21 (d, 5.2) | 31.5 | 1.22 (d, 5.0) | 31.5 | 0.96 (d, 5.1) | 31.6 | 1.20 (d, 5.4) |
| 15 | 26.8 | / | 26.8 | / | 22.2 | / | 26.9 | / |
| 16 | 11.5 | 1.19 (s) | 11.6 | 1.20 (s) | 14.9 | 1.04 (s) | 11.5 | 1.18 (s) |
| 17 | 69.7 | 4.24 (d, 11.4)  4.28 (d, 11.4) | 69.7 | 4.24 (d, 11.3)  4.29 (d, 11.3) | 22.8 | 1.18 (s) | 69.3 | 4.17 (d, 11.3)  4.21 (d, 11.3) |
| 18 | 18.9 | 0.96 (d, 6.1) | 18.9 | 0.97 (d, 6.6) | 18.3 | 1.00 (d, 6.3) | 18.8 | 0.96 (d, 5.9) |
| 19 | 10.0 | 1.58 (dd, 2.9, 1.2) | 10.0 | 1.58 (bd, 1.8) | 9.4 | 1.58 (dd, 2.6, 1.1) | 10.0 | 1.58 (dd, 2.9, 1.3) |
| 20 | 67.8 | 3.58 (dd, 12.8, 5.3)  3.63 (dd, 12.8, 5.3) | 67.9 | 3.62 (d, 12.9)  3.68 (d, 12.9) | 67.5 | 3.70 (d, 12.8)  3.76 (d, 12.8) | 67.8 | 3.59 (d, 12.8)  3.64 (d, 12.8) |
| 20-OH | - | 0.63 (t, 5.3) |  | / | - | / | - | / |
| 1’ | 175.7 | / | 175.7 | / | 175.0 | / | 175.7 | / |
| 2’ | 34.6 | 2.07^b^ | 34.6 | 2.08^b^ | 34.1 | 2.07 (t, 7.6) | 34.6 | 2.10 (m) |
| 3’ | 25.0 | 1.48 (m) | 25.0 | 1.50 (m) | 24.5 | 1.51 (m) | 25.0 | 1.51 (m) |
| 4’ | 29.3 | 1.14^b^ | 29.4 | 1.16^b^ | 28.7 | 1.16^b^ | 29.57 | 1.18^b^ |
| 5’ | 29.58^a^ | 1.14-1.23 | 30.03^a^ | 1.16-1.32 | 29.10^a^ | 1.16-1.22 | 29.36^a^ | 1.16-1.27 |
| 6’ | 29.65^a^ | 1.14-1.23 | 30.03^a^ | 1.16-1.32 | 28.98^a^ | 1.16-1.22 | 29.64^a^ | 1.16-1.27 |
| 7’ | 29.74^a^ | 1.14-1.23 | 29.82^a^ | 1.16-1.32 | 28.90^a^ | 1.16-1.22 | 29.74 | 1.16-1.27 |
| 8’ | 32.2 | 1.23^b^ | 29.78^a^ | 1.16-1.32 | 31.6 | 1.22^b^ | 32.2 | 1.24^b^ |
| 9’ | 23.1 | 1.30 (m) | 29.61^a^ | 1.16-1.32 | 22.4 | 1.29 (m) | 23.1 | 1.30 (m) |
| 10’ | 14.0 | 0.91 (t, 7.0) | 32.3 | 1.28^b^ | 13.7 | 0.91 (t, 7.1) | 14.3 | 0.91 (t, 7.3) |
| 11’ | - | - | 23.1 | 1.30 (m) | - | - | - | - |
| 12’ | - | - | 14.4 | 0.92 (t, 7.3) | - | - | - | - |
| 1” | 167.5 | / | 167.5 | / | - | - | 175.9 | / |
| 2” | 129.2 | / | 129.2 | / | - | - | 41.3 | 2.33 (qdd, 7.0, 7.0, 7.0) |
| 3” | 136.9 | 6.99 (qq, 7.2, 1.4) | 136.9 | 6.98 (qq, 7.1, 1.3) | - | - | 27.2 | 1.40 (dqd, 13.6, 7.0, 7.0)  1.72 (dqd, 13.6, 7.0, 7.0) |
| 4” | 14.0 | 1.38 (dq, 7.2, 1.0) | 14.0 | 1.38 (d, 7.1) | - | - | 11.8 | 0.88 (t, 7.0) |
| 5” | 12.2 | 1.84 (m) | 12.2 | 1.84 (bs) | - | - | 16.9 | 1.12 (d, 7.0) |

^a^Assignments within a column are interchangeable. ^b^Multiplicity not determined due to overlapping signals/chemical shifts determined from 2D data.

**Table S2.** Determination of relative levels of namushens in crude Mukungulu batches A and B.

**Table S3.** Side effects of Mukungulu treatment in CD-1 mice. 3 female and 3 mice each were injected with a single dose of 0.125, 1.25, or 12.5 mg/kg and monitored. Values in parenthesis denote number of mice with observed behavior.

**Table S4.** pVL data in ART-suppressed, HIV-infected BLT humanized mice treated with PBS (left) or 5 mg/kg Mukungulu (right). In each table, top row denotes mouse number. pVL is described in log10 copies per mL plasma. UD, undetectable.

^
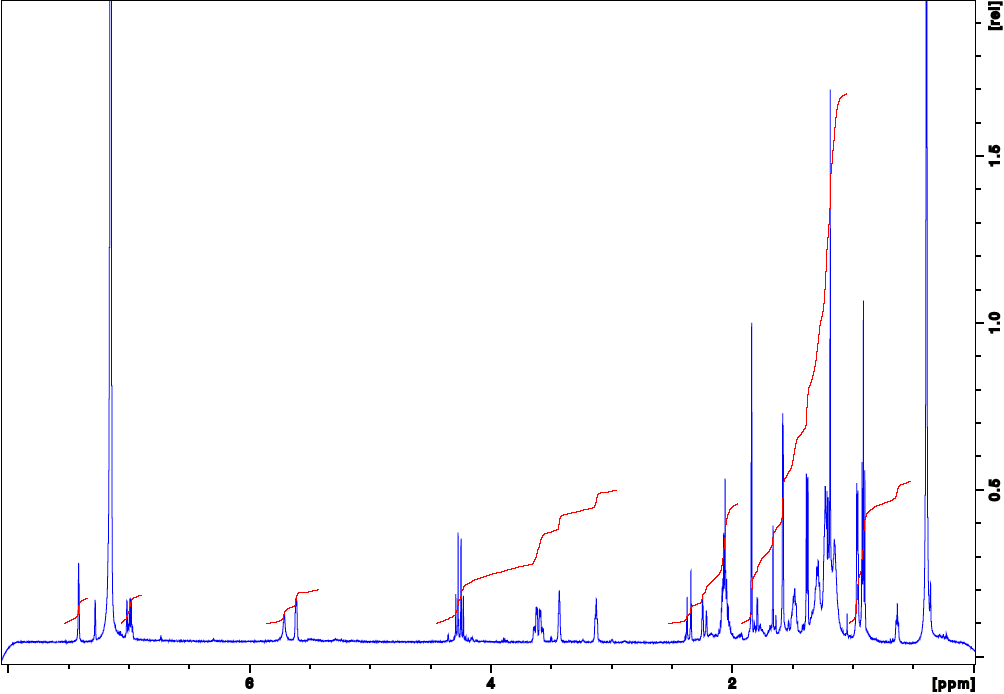
^

**Figure S1.** ^1^H NMR Spectrum of namushen-3 recorded at 600 MHz in C_6_D_6_.

**
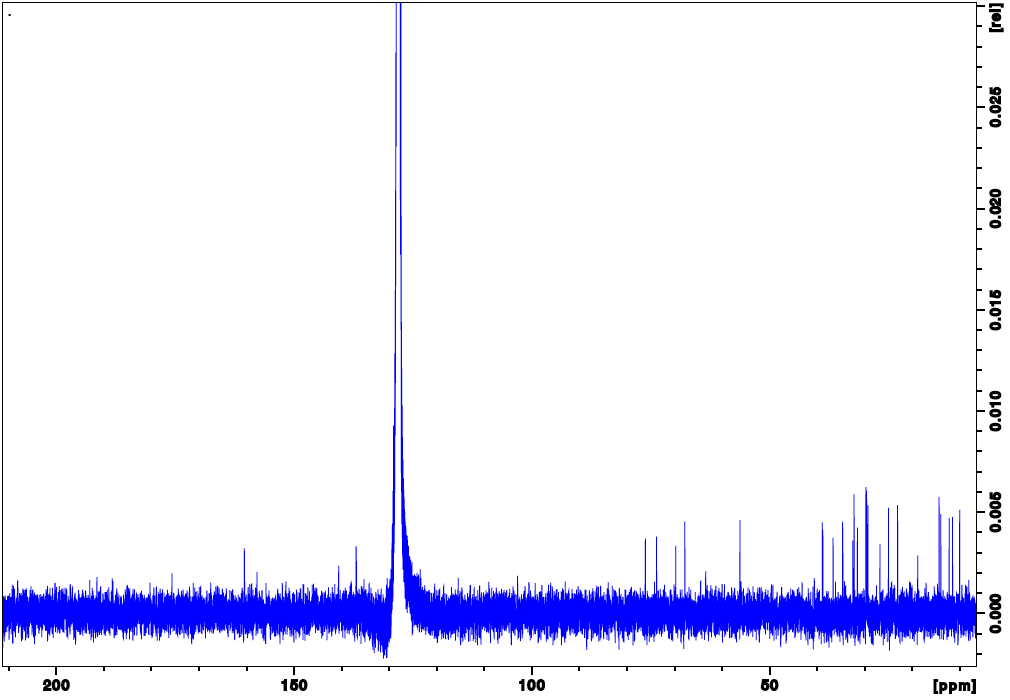
**

**Figure S2.** ^13^C NMR Spectrum of namushen-3 recorded at 150 MHz in C_6_D_6_.

_
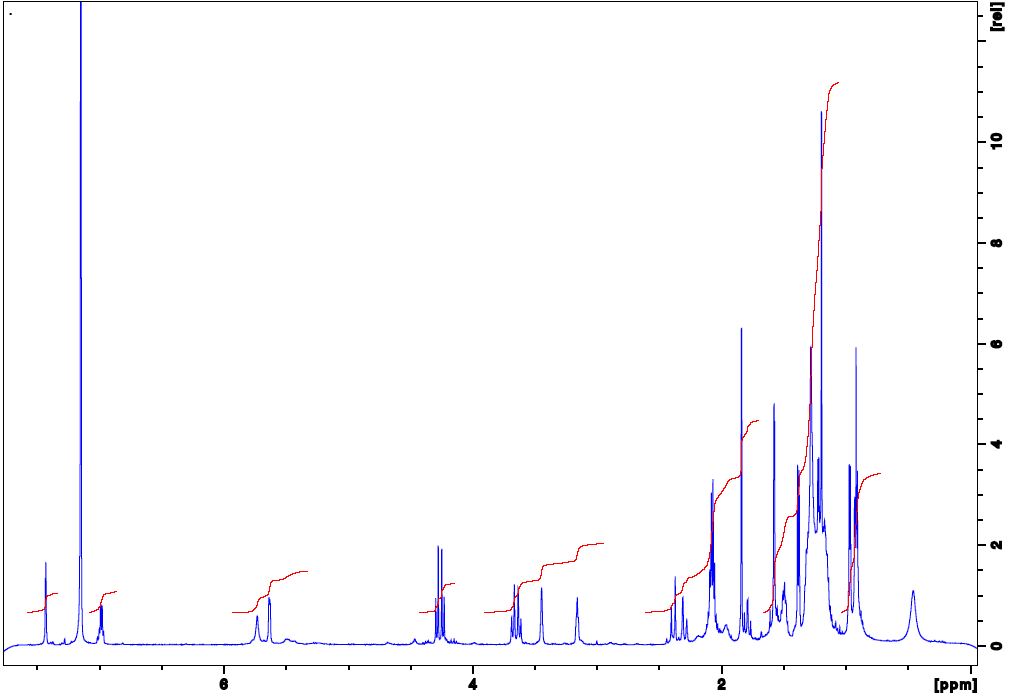
_

**Figure S3.** ^1^H NMR Spectrum of namushen-4 recorded at 600 MHz in C_6_D_6_.

^
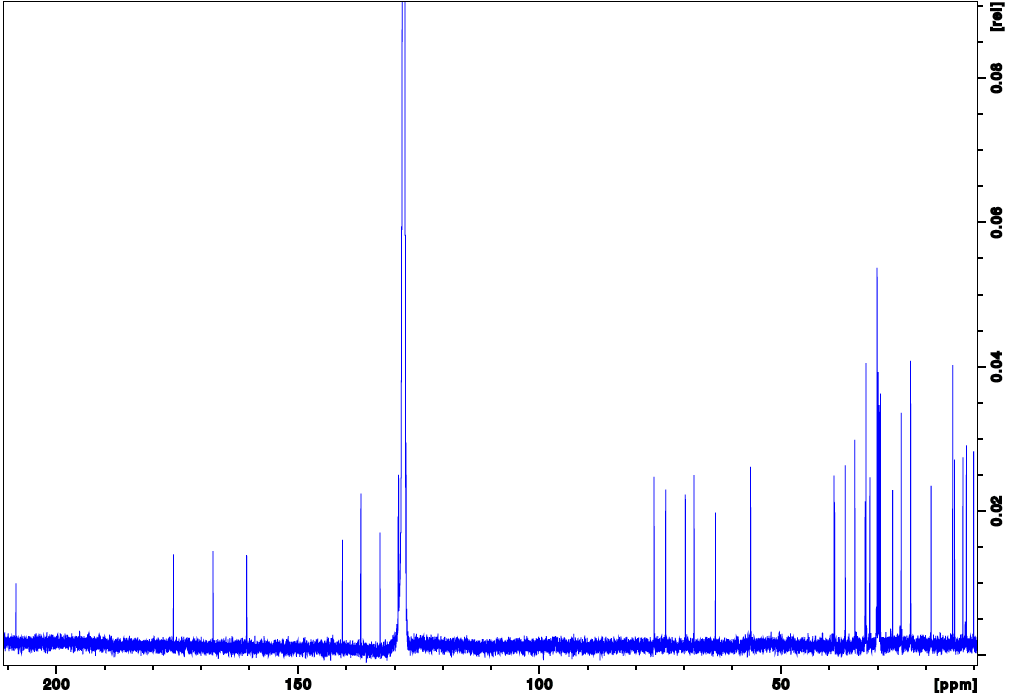
^

**Figure S4.** ^13^C NMR Spectrum of namushen-4 recorded at 150 MHz in C_6_D_6_.

**
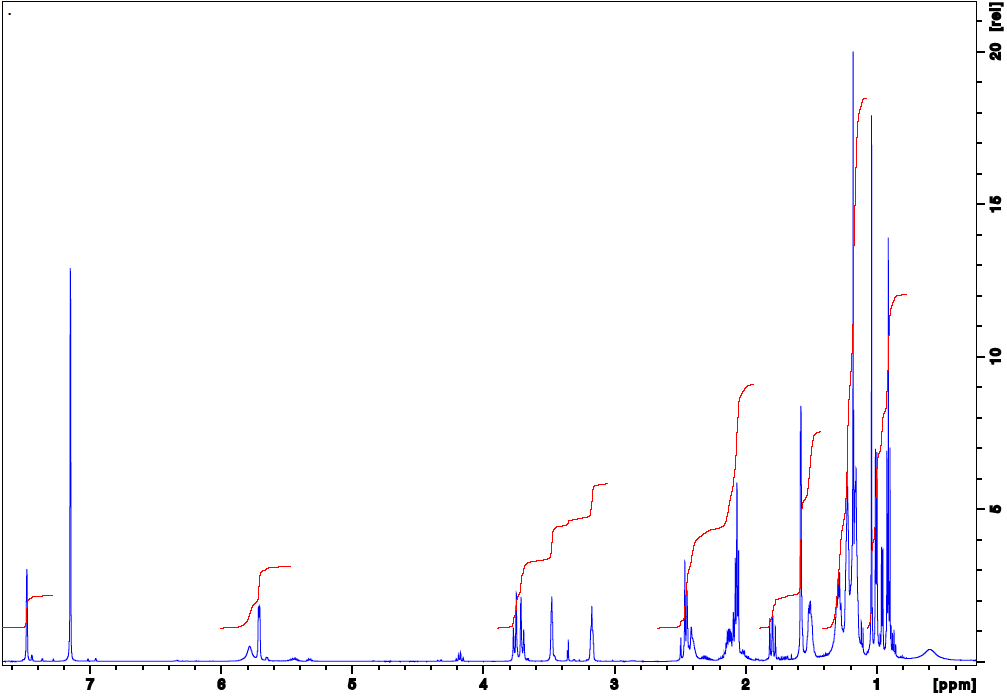
**

**Figure S5.** ^1^H NMR Spectrum of namushen-5 recorded at 600 MHz in C_6_D_6_.


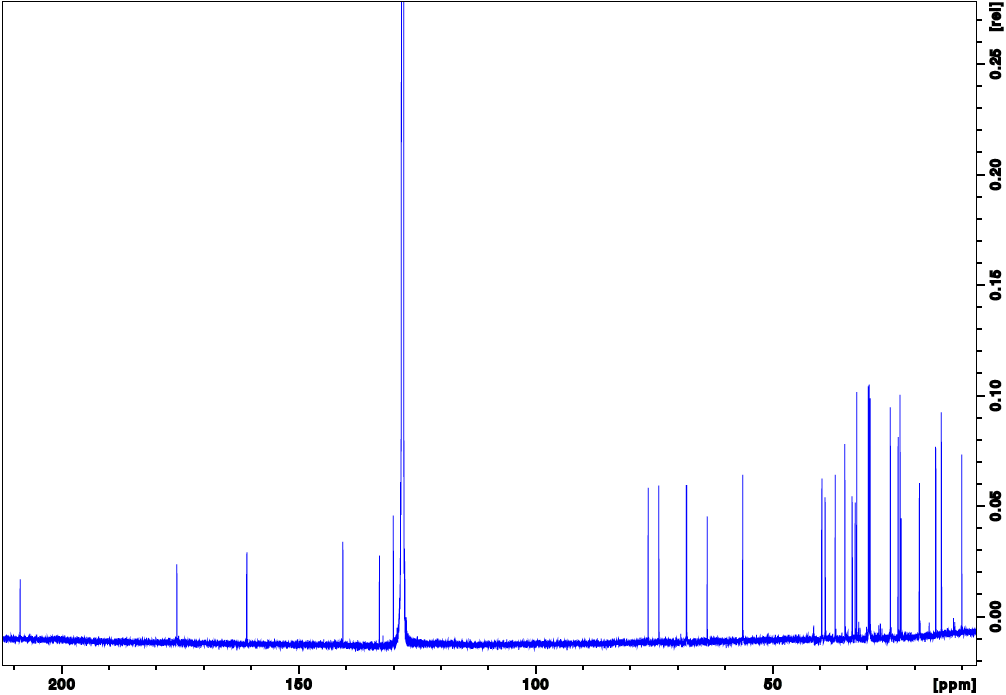

**Figure S6.** ^13^C NMR Spectrum of namushen-5 recorded at 150 MHz in C_6_D_6_.

**Figure S7.** Correlations of viral protein levels in PBMC pellets (left) and supernatants (right) following 72 hours treatment with anti-CD3/CD28 (top) or 1 µg/mL Mukungulu (bottom), as measured by simoa, relative to percent CD3+ CD4+ cells.

**Figure S8.** Correlations of viral protein levels in PBMC pellets following 72 hours treatment with anti-CD3/CD28 (top) or 1 µg/mL Mukungulu (bottom), as measured by simoa, relative to total, intact, total defective, 5’-defective, or 3’-defective provirus in CD4+ T-cells, as measured by IPDA. Unfilled shapes denote gag-p24 protein below the LOD which is annotated here at 0.005 pg/mL.

**Figure S9.** Correlations of viral protein levels in PBMC supernatants following 72 hours treatment with anti-CD3/CD28 (top) or 1 µg/mL Mukungulu (bottom), as measured by simoa, relative to total, intact, total defective, 5’-defective, or 3’-defective provirus in CD4+ T-cells, as measured by IPDA. Unfilled shapes denote gag-p24 protein below the LOD which is annotated here at 0.005 pg/mL.
